# The Power of Unanimity

**Published:** 1902-12-06

**Authors:** 


### THE POWER OF UNANIMITY.

Medical practitioners seldom tire of reiterating the complaint that their profession is without voice and powerless in the councils of the nation. Nor is this complaint entirely without foundation. Truly the public display a curious readiness to accept the sayings of well-known professional leaders as final and indisputable verities, even though they deal with subjects on which their authors are by no means great anthorities. But this blind confidence in *ex cathedrâ* utterances does not extend very deeply. It is but a phase of that hero or notoriety worship which is such a curious characteristic of modern times, for ready as people may show themselves to accept the opinions of those leaders in medicine who bask in the sunshine of newspaper publicity, this is largely because they are well-known personages—not because they are doctors. The preaching of the ordinary practitioner is still as that of a voice crying in the wilderness, and it is perhaps not to be wondered at that we bewail the stupidity of those who will not listen to us. Yet should not the ignominious position to which medicine is too often relegated be regarded as implying a reproach to its professors? and is not the contempt with which its teachings are too often received due to the fact that such teachings are frequently not only lacking in unanimity but actually bristling with differences?

In the political constitution under which we dwell, a profession, which numbers nigh on 30,000 members, every one of whom each day of his life comes in contact with say on an average a score of people who are willing in certain matters to follow his advice, ought to have an influence and a power of moulding opinion second only to that exercised by the Press. Speaking recently at Brighton, Dr. Gordon Dill pointed out with great force and appropriateness that we—*i.e.* the mass of medical practitioners—are the moulders of public ideas upon medical matters, and that it only requires time and publicity for any theory upon which medical men are agreed to become an axiom of the man in the street, and consequently the precursor of legislative enactments. As then any measure touching the public health will, under our present system of government have a chance of success just in proportion to the number of electors who are persuaded that their personal well-being or that of their families is at stake, the present indifference of the public in regard to many matters of great sanitary importance does in fact lie at our own door—at the door of those who ought to be its teachers. Each one of us is, or ought to be, a teacher in his own particular surrounding, and responsible for that moulding of public opinion on which the making of law depends. But to be of effect our teaching must be unanimous—agreed in theory, and preaching the same doctrine of progress—and this is where we fail. The differences of doctors have for generations been made a subject of ridicule by scoffers. They have their root however in conditions, some of which are unavoidable. The rapid accretion of knowledge, the constant progress made in medical arts and science, the frequent changes in theory which are caused by the discovery of new facts, all tend not merely to establish varied points of view from which the same conditions may be regarded and thus to give rise to varied opinions in regard to them, but to lead practitioners to arrive at very different conclusions according to the date of the knowledge upon which they found their arguments. The former of these causes of differences among doctors can never be eliminated, being common to all progressive sciences, but the latter ought to be continually diminished by the growing rapidity with which newly-gained knowledge becomes diffused throughout the rank and file of the profession. Our hopes of unanimity on professional matters must, as it seems to us, be chiefly based upon that readiness to continue post-graduate study throughout their whole professional lives, which is every year becoming more and more apparent even among senior practitioners. Such unanimity as would arise from an approximate equality of knowledge would go far to render our teaching respected by the people and powerful in its influence for the public good.

